# Citrate Anticoagulation for CRRT in Children: Comparison with Heparin

**DOI:** 10.1155/2014/786301

**Published:** 2014-08-03

**Authors:** Sara Nicole Fernández, Maria José Santiago, Jesús López-Herce, Miriam García, Jimena Del Castillo, Andrés José Alcaraz, Jose María Bellón

**Affiliations:** ^1^Pediatric Intensive Care Unit, University General Hospital Gregorio Marañón, Calle Doctor Castelo 47, 28009 Madrid, Spain; ^2^Statistics, Preventive Medicine and Quality Service, University General Hospital Gregorio Marañón, Complutense University, Madrid, Spain

## Abstract

Regional anticoagulation with citrate is an alternative to heparin in continuous renal replacement therapies, which may prolong circuit lifetime and decrease hemorrhagic complications. A retrospective comparative cohort study based on a prospective observational registry was conducted including critically ill children undergoing CRRT. Efficacy, measured as circuit survival, and secondary effects of heparin and citrate were compared. 12 patients on CRRT with citrate anticoagulation and 24 patients with heparin anticoagulation were analyzed. Median citrate dose was 2.6 mmol/L. Median calcium dose was 0.16 mEq/kg/h. Median heparin dose was 15 UI/kg/h. Median circuit survival was 48 hours with citrate and 31 hours with heparin (*P* = 0.028). 66.6% of patients treated with citrate developed mild metabolic alkalosis, which was directly related to citrate dose. There were no cases of citrate intoxication: median total calcium/ionic calcium index (CaT/I) of 2.16 and a maximum CaT/I of 2.33, without metabolic acidosis. In the citrate group, 45.5% of patients developed hypochloremia and 27.3% hypomagnesemia. In the heparin group, 27.8% developed hypophosphatemia. Three patients were moved from heparin to citrate to control postoperatory bleeding. In conclusion citrate is a safe and effective anticoagulation method for CRRT in children and it achieves longer circuit survival than heparin.

## 1. Introduction

Continuous renal replacement therapies (CRRT) are the most commonly used renal replacement therapies in critically ill patients with acute renal failure [1, 2]. Efficacy of these therapies is directly related to the running time of the circuit. The most common complication is early clotting of the filter. Prolonged circuit survival is very important, as filter clotting can have several negative effects: decreased efficacy of treatment, increased blood loss (especially in smaller patients), haemodynamic instability during reconnection, and, of course, increased costs [1, 2].

Several anticoagulation protocols have been used in CRRT. Heparin has been normally used for anticoagulation in Europe. Regional anticoagulation with citrate has been proposed for critically ill patients for whom systemic anticoagulation is discouraged. Regional citrate anticoagulation (RCA) is associated with a lower incidence of bleeding [2]. It is a safer alternative than heparin for high risk of bleeding patients and a good alternative for patients with heparin-induced thrombopenia (HIT). In addition, available scientific evidence suggests that RCA prolongs circuit survival [2].

However, few observational studies have been published about citrate anticoagulation in pediatric patients [3, 4] and no one has compared the efficacy of heparin and citrate for anticoagulation in critically ill children on CRRT. This is the objective of our study.

## 2. Material and Methods

A retrospective comparative cohort study based on a prospective observational registry was conducted including critically ill children undergoing CRRT treated with citrate and heparin. The study was approved by the local Institutional Review Board. We included all patients treated with citrate. To improve statistical power each patient treated with citrate was coupled with two patients treated with heparin that used the same type of filter. Patients requiring ECMO at the time of hemofiltration were excluded.

**Figure 1:**
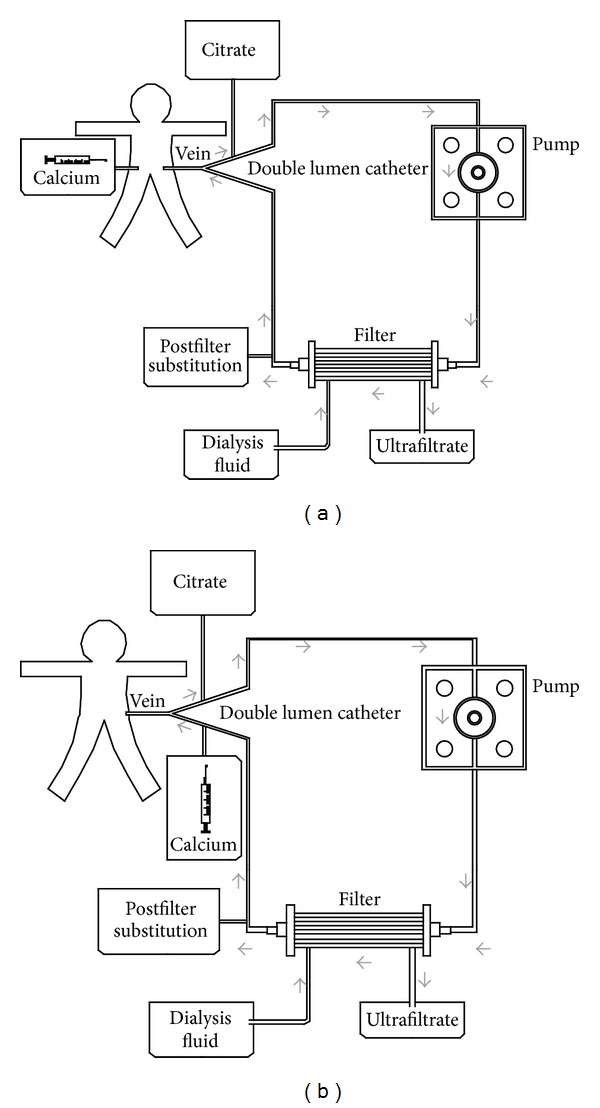
Regional citrate anticoagulation figure. (a) Calcium administration in a different central vein; (b) calcium administration in the circuit return.

A Prismaflex device (Gambro, Spain) was used for CRRT in all patients. Catheters between 4 F and 11 F were used depending on the age and weight of the child, using the same criteria for patients treated with citrate and heparin anticoagulation: 5 F dual-lumen catheters were used in children weighing less than 5 kg, 6.5 F dual-lumen catheters in patients between 5 and 15 kg, and 8 F to 11 F catheters in patients weighing over 15 kg. The femoral vein was catheterized in 83% of patients and the jugular vein in 17% of them. Hollow-fibre haemofilters were used, depending on the body surface area of the patient and on the pump employed. Polyarylethersulfone HF20 0.2 m^2^ filters (Gambro) were used in children weighing less than 5 kg, M60 (Gambro) 0.6 m^2^ in patients weighing between 5 and 35 kg, and M100 (Gambro) 0.9 m^2^ in children weighing over 35 kg. In both anticoagulation methods, blood flow was programmed between 3 and 6 mL/kg/hour according to patient weight, catheter size, and filter surface. The composition of the solutions used is presented in Table 1. The commercially prepared citrate solution (Prismocitrate, Gambro, Spain) was administered prefilter ahead of the blood pump (Figure 1). Two different citrate solutions were used: 12 mmol/L in the first four patients and 18 mmol/L in the other patients. An initial citrate concentration of 2.5 mmol/L was used for a target postfilter ionized calcium of 0.25 to 0.35 mmol/L. Calcium-free dialysis solutions were used (PrismOcal, Gambro, Spain). To avoid deaeration chamber coagulation a low-flow postfilter infusion with a calcium-free solution was also used (Clear-Flex, Baxter International).

Citrate effect was neutralized using a continuous calcium infusion, calcium gluconate 10% 50/50 with glucose 5% in a 50 mL syringe (0.22 mEq/mL; 0.11 mmol/mL). An initial infusion rate of 0.5 mL/kg/h (0.11 mEq/kg/h or 0.06 mmol/kg/h) was used to maintain ionized calcium blood levels between 1.1 and 1.3 mmol/L (samples were taken every hour for the first four hours and every eight hours afterwards; patient calcium levels were also checked one hour after any change in blood flow, citrate concentration, or dialysis). Calcium was initially infused through the return line of the circuit (in the first 8 patients), but an increase of catheter malfunction was observed, so the protocol was changed and calcium infusion was administered through a different central venous line (according to some protocols in adult patients [5]), after which the incidence of catheter malfunction returned to previous rates (Figure 1(a)). Only when a separate central I.V. was not available, did we administer a calcium infusion through the return line of the circuit (Figure 1(b)). In the heparin anticoagulation protocol a 50 UI/kg bolus was administered during the connection along with a continuous heparin sodium of 10–20 UI/kg/h to achieve a postfilter activated clotting time (ACT) between 160 and 180. Phoxilium was used as a substitution and dialysis solution, except in the case of hyperkaliemia (*K* > 5.5) or hyperphosphatemia (*P* > 7 mg/dL). In those cases Hemosol BO was used. Substitution flow was programmed initially at 20 mL/kg/hour and dialysis flow was added according to the needs of the patient.

Administered dose of heparin and citrate, mean survival of filter, and secondary effects were recorded. Efficacy, measured as circuit survival, and secondary effects of heparin and citrate were compared. Citrate accumulation was suspected in the case of metabolic acidosis and low ionized calcium levels with an increase in the total calcium/ionic calcium index (CaT/I) ≥ 2.5 [6].

Data are expressed as medians and interquartile ranges (IQR). A Kaplan-Meier survival analysis was performed so as to estimate the circuit lifetime probability according to the different anticoagulation modalities.

## 3. Results

Between January 2011 and February 2013 regional citrate anticoagulation was used in 12 patients. The last 24 patients that used the same type of filter but used heparin anticoagulation on CRRT were analysed. Most patients in both groups were in the postoperative period of cardiac surgery (83.3% in citrate group and 58.3% in heparin group). Other diagnoses were postoperative period of abdominal surgery, sepsis, and tumor lysis syndrome. The clinical characteristics of the patients are shown in Table 2. The median age was 36 months in citrate group and 33 months in the heparin group (*P* = 0.882).

Most patients in both groups had a femoral access (83% in citrate group and 71% in heparin group) and the most frequently used catheter was the 6.5 FR. There were no differences in inotropic score and risk of mortality scores between the two groups (Table 2). Mortality rate was the same in both groups: 25%. The median stay in the PICU for those who survived was 17 days in the citrate group and 22 days in the heparin group (no significant difference). Characteristics of CRRT in both groups are shown in Table 3.

In the citrate group 6 patients used 23 HF20 (0.2 m^2^) filters; in heparin group 12 patients used 59 HF20 filters. The M60 (0.6 m^2^) was used in 4 patients in the RCA group (6 filters) and in 8 patients in heparin group (29 filters). Finally, the M100 (0.9 m^2^) was used in 2 citrate patients (5 filters) and in 4 heparin patients (8 filters).

Median treatment duration was not statistically different (*P* = 0.736) between both groups: 155 hours for the citrate group and 166 hours for the heparin group. The median dose of citrate was 2.6 mmol/L (IQR 2.3–2.9), with a median flow of 67 mL/kg/h for the Prismocitrate 10/2 solution and 28.1 mL/kg/h for the Prismocitrate 18/0 solution. The final citrate dose was 2.9 mmol/L (IQR 2.5–3.2). Calcium was initially infused through the return line of the circuit (in the first 8 patients), but an increase of catheter malfunction was observed, so the protocol was changed and calcium infusion was administered through a different central venous line. Median final calcium dose was 0.16 mEq/kg/h (IQR 0.13–0.22). A median of 2 changes (IQR1-5.5) in calcium doses and 13 hours (IQR 10.25–36) were necessary to achieve the final calcium dose. Infants (<12 kg) required slightly higher doses of calcium than older children (0.17 mEq/kg/h, IQR 0.16–0.22 versus 0.13 mEq/kg/h, IQR 0.10–0.15).

The median anticoagulant dose in the heparin anticoagulation group was 15 UI/kg/hour (IQR 12–25 UI/kg/hour). Total CRRT dose (sum of substitution, dialysis, citrate, and extraction flows) per kilogram was higher in the citrate group, but the difference was not statistically significant (69 versus 59 mL/kg/hour; *P* = 0.154).

**Figure 2:**
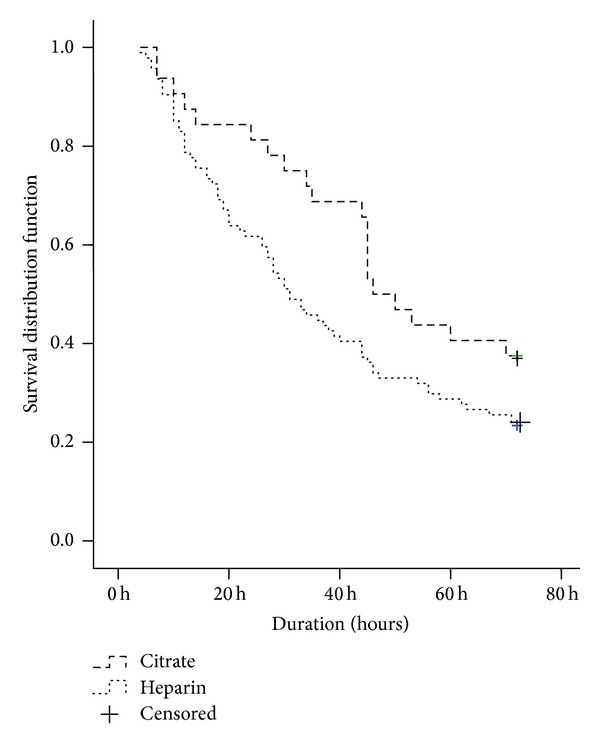
Kaplan-Meier circuit survival curve comparing citrate and heparin.

Median circuit survival was 48 hours (IQR 31.0–93.7) in the citrate group and 31.0 hours (IQR 15.5–71.0) in the heparin group (*P* = 0.028). In Figure 2 the Kaplan-Meier circuit survival curve comparing citrate and heparin is shown.

The 12 mmol/L citrate solution had a median filter duration time of 60 hours (IQR 45–101) which was slightly higher, but not statistically significant (*P* = 0.075), than the 18 mmol/L citrate solution at 44 hours (IQR 24–84). The causes of circuit replacement in the citrate group compared to the heparin group are shown in Table 4.

Clotting was the main cause of circuit change in heparin filters (76.4% versus 18.8% in the citrate group; *P* < 0.01). On the other hand, regularly scheduled filter change was more common (31.3%) with citrate anticoagulation than with heparin (11%; *P* < 0.01). 66.6% of patients treated with citrate developed mild metabolic alkalosis and it was directly related to citrate dose (*r* = 0.665, *P* = 0.036). In comparison with heparin, citrate anticoagulation produced more hypochloremia (45.5% versus 0%; *P* < 0.01) and hypomagnesemia (27.3% versus 0%; *P* = 0.045) than heparin. In the heparin group there was more hypophosphatemia (27.8% versus 0%; *P* = 0.06).

There were no cases of citrate accumulation (high anion gap metabolic acidosis and low ionized calcium levels). Median total calcium/ionic calcium index (CaI) was 2.16 (IQR 2.08–2.25), and maximum CaI was 2.33, without metabolic acidosis.

**Figure 3:**
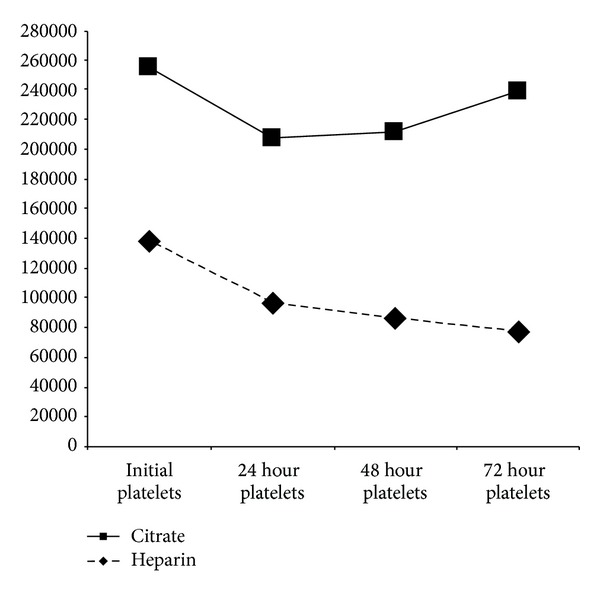
Platelet evolution.

The evolution of platelet levels in both groups during treatment is presented in Figure 3.

Both groups experienced a nonsignificant drop in platelet levels in the first 24 hours, but afterwards there was a progressive decrease throughout treatment in the heparin group (median initial platelets: 138.000; IQR 74.000–194.500; median platelets at 72 hours: 77.000; IQR 32.000–111.000; *P* = 0.09), whereas platelets remained stable in the citrate group (median initial platelets: 117.000; IQR 75.000–191.000; median platelets at 72 hours: 161.000; IQR 38.000–257.000; *P* = 0.602).

Three patients were moved from heparin to citrate to control postoperatory hemorrhagic complications which resulted in ceased bleeding.

## 4. Discussion

The ideal anticoagulant should act selectively in the circuit with minimal effects on patient homeostasis, it should be safe, readily available, consistently delivered, easily monitored, and reversible, and it should provide prolonged filter lifetime and good cost-effectiveness. Citrate is an anticoagulant by virtue of its ability to bind with calcium. Citrate administered before the pump reacts with free calcium of the blood; they are bound together, and thus free calcium levels are lowered. Ionic calcium returns to normal levels by three mechanisms: the citrate molecule is small (258.06 g/mol), so it passes through the filter and is cleared by convection and diffusion, the effect is neutralized with the administration of calcium after the filter to ensure normal free calcium levels in the patient, and the remaining citrate in the return line is diluted in the systemic circulation and is quickly metabolized, mostly in the liver.

The lack of a systemic anticoagulation effect is especially important in patients requiring CRRT in the postoperative period of cardiac surgery, with a high risk of bleeding complications [5]. In addition, citrate does not induce thrombopenia, which also reduces bleeding risk.

No randomized controlled trials and very few observational studies have been carried out in pediatric patients on CRRT treated with citrate [3, 4]. Only 6 randomized controlled trials comparing citrate and heparin anticoagulation have been published in adult patients [7–12]. They do not include high risk bleeding patients for ethical reasons. Zhang and Hongying performed a systematic review of these randomized trials [2]. Our study is the first in children that compared the effect of citrate and heparin in critically ill children. Comparing our results with those published in adult patients, the median dose of citrate is lower (2.59 mmol/L versus 3-4 mmol/L [2, 5]) and the median dose of calcium is higher (0.16 mEq/kg/h versus only 1.19 mEq/h in the study of Morabito [5]).

Initial infusion rate of calcium had to be changed because infants require a higher calcium dose than older patients. Now we administer 0.22 mEq/kg/h (0.11 mmol/kg/h) in infants and 0.11 mEq/kg/h (0.06 mmol/kg/h) in older children.

The median circuit survival was significantly higher for citrate than for heparin with a mean difference of 17 hours (*P* = 0.028), which is consistent with the analysis of randomized trials in adults, showing a mean difference of 23 hours (IC 95% 0.45–45.61) [2]. Clotting was the main cause of circuit change in heparin filters (76%) as opposed to only 18% in the citrate group. On the other hand, scheduled filter change was more common with citrate than with heparin. Technical issues occurred like incorrect volume balance caused by movement of the device that caused the pump stop (8 times) and software crashes which required that the pump be rebooted (3 times) (which happened when the Prismaflex machines and software were changed and updated). Initially we programmed the same blood flow in both methods, but later we realized that a lower blood flow was required with citrate anticoagulation, obtaining greater circuit survival with lower substitution rate and thus with less citrate flow.

The incidence of bleeding was much lower in patients treated with citrate than with heparin (0% versus 10.3%) [13] which is consistent with the meta-analysis that showed an overall risk ratio with citrate of 0.28 (95% CI 0.15–0.50) [2]. In addition thrombopenia has two major disadvantages: it increases bleeding risk and increases the need for blood product transfusions. Platelet count remained stable in patients treated with citrate, as opposed to heparin patients, who developed significant thrombopenia.

As a result, healthcare-related costs could be lowered as patients require fewer blood product transfusions and fewer circuits are wasted.

The most common metabolic complication related to citrate is metabolic alkalosis because citrate is metabolized in the liver, yielding three molecules of bicarbonate for every molecule of citrate. In our study, metabolic complications were mild in all patients. Adjustments in dialysis and replacement solutions were required because of hypochloremia in 45.5% of patients and hypomagnesemia in 27.3% of patients. Low chloride levels were observed in most of the patients because Prismocitrate solutions contain only 86 mmol/L of chloride. No cases of significant hypo- or hypercalcaemia, metabolic acidosis, liver failure, or citrate intoxication were observed. Citrate intoxication has been reported in patients with liver function impairment, causing citrate to accumulate in the systemic circulation and leading to metabolic acidosis and hypocalcaemia (low ionized calcium levels, as total calcium may remain normal). Nevertheless, some studies show that it is safe to use citrate in patients with mild-moderate liver dysfunction [14]. Our results are comparable to those in the meta-analysis of Zhang and Hongying [2], where metabolic derangements were mild without significant clinical consequences. Thus, metabolic complications in patients treated with citrate should not be considered a drawback, since they are mild and easily resolved.

Our study has several limitations. Although it is the first comparison study in children between citrate and heparin, it was not a randomized study and it only included a small number of patients. It describes our initial experience with citrate (which partly explains the incidence of technical problems with the circuit). Another limitation is that two types of citrate solutions were used (12.0 and 18.0 mmol/L).

## 5. Conclusion

Citrate is a safe and effective anticoagulation method for continuous renal replacement therapies in children, as it appears to prolong circuit survival with a lower incidence of clotting. The other advantage of citrate is its lower incidence of bleeding complications as it does not have systemic anticoagulation effects and it does not induce thrombopenia.

The main disadvantages of citrate are the need for close monitoring of electrolyte and acid-base equilibrium, an additional venous central line for calcium infusion, and a well-trained nursing staff.

## Figures and Tables

**Table 1:** Composition of the solutions.

	PrismOcal	PrismOcal B22	Prismocitrate		Hemosol B0	Clear-flex D6	Phoxilium
			10/2	18			
Volume	5000 mL	5000 mL	5000	5000	5000 mL	3000 mL	5000 mL
Na mmol/L	140	140	136	140	140	140	140
K mmol/L	0	4	0	0	0	0	4
Cl mmol/L	106	120	106	86	109.5	100	115.9
Ca mmol/L	0	0	0	0	1.75	0	1.25
Mg mmol/L	0.5	0.75	0	0	0.5	0	0.6
CO3H	32	22	0	0	32	40	30
Lactate	3	3	0	0	3	0	0
Glucose mg/dL	0	109.8	0	0	0	0	0
Phosphate mmol/L	0	0	0	0	0	0	1.2
Citrate/citric acid mmol/L			10/2	18/0			
Administration route	Dialysis	Dialysis	IV PBP∗	IV PBP∗	Postfilter substitution	Pre- or postfilter substitution	Postfilter substitution

*PBP: placed in a pump before blood pump.

**Table 2:** Clinical characteristics of children treated with heparin and citrate.

	Citrate	Heparin	*P*
	Median (IQR)	Median (IQR)	
Age (months)	36.0 (5.6–103.7)	33.0 (5.6–84.0)	0.882
Weight	13.4 (6.8–28.8)	9.8 (6.3–19.8)	0.585
PRISM (% of mortality)	12.6 (3.4–20.8)	10.0 (6.4–31.4)	0.716
PIM (% of mortality)	16.6 (6.9–22.6)	10.6 (5.5–20.4)	0.431
MOF	3 (3–3.75)	3 (3-4)	0.681
PELOD (% of mortality)	11 (11–21)	21 (12.25–22)	0.145
Lactic acid	2.5 (1.3–6.8)	1.4 (1.0–2.4)	0.075
Inotropic score	40 (18–54)	38 (4–53)	0.540
Mechanical ventilation	91.7%	81.8%	0.635
Mortality	25%	25%	1.000

IQR: interquartile range. MOF: multiorgan failure.

**Table 3:** Characteristics of CRRT in children treated with heparin and citrate.

	Citrate	Heparin	*P*
	Median (IQR)	Median (IQR)	
Blood flow (mL/min)/weight (kg)	3.2 (2–3.8)	5.0 (3.8–5.6)	0.008
Citrate dose (mmol/L)	2.6 (2.3–2.9)	—	—
Citrate flow (citrate 12 mmol/L) (mL/kg/h)	67 (50.9–73.7)	—	—
Citrate flow (citrate 18 mmol/L) (mL/kg/h)	28 **(**21.3–30.8)	—	—
Final citrate dose (mmol/L)	2.9 (2.5–3.2)	—	—
Heparin (UI/kg/h)	—	15 (12–25)	—
Extraction rate (mL/h)	75 (50–97.5)	60 (50–90)	0.526
Dialysis (mL/h)	325 (50–600)	400 (200–750)	0.289
Postfilter substitution (mL/h)	50 (0–50)	300 (140–500)	0.026
Total CRRT doses (mL/kg/h)	69 (52–85)	59 (44–70)	0.154
CRRT duration (h)	155 (92–204)	166 (92–271)	0.736

IQR: interquartile range.

**Table 4:** Causes of circuit replacement.

Causes	Citrate (*n*; %)	Heparin (*n*; %)
Clotting	6 (18.8%)	68 (76.4%)
Scheduled/medical procedures	10 (31.3%)	10 (11.2%)
Catheter malfunction	5 (15.6%)	10 (11.2%)
Technical issues∗	11 (34.3%)	1 (1.1%)

*Incorrect scale balance caused the pump to stop and the software to crash which required that the pump be rebooted.
